# Acute Ischemic Gangrene of the Rectosigmoid Colon in a Patient With Rectal Cancer in the "Watch-and-Wait" Protocol

**DOI:** 10.7759/cureus.14998

**Published:** 2021-05-13

**Authors:** Vusal Aliyev, Suha Goksel, Oktar Asoglu

**Affiliations:** 1 General and Colorectal Surgery, Maslak Acibadem Hospital, Istanbul, TUR; 2 Pathology, Maslak Acibadem Hospital, Istanbul, TUR; 3 General and Colorectal Surgery, Bogazici Academy for Clinical Sciences, Istanbul, TUR

**Keywords:** ischemic proctosigmoiditis, rectal cancer, total neoadjuvant treatment, complete clinical response, ‘’watch-and-wait’’

## Abstract

Acute rectal ischemia is a rare entity because the rectum has abundant blood supply from the inferior mesenteric, internal iliac, internal pudendal, and marginal artery with rich collaterals.

We present a case of an acute ischemic proctosigmoiditis with a history of rectal cancer who completely recovered after total neoadjuvant treatment and was in the “watch-and-wait” protocol. Urgent laparoscopic low anterior resection and protective ileostomy were performed. Causes of acute rectosigmoid ischemia include old age, diabetes, atherosclerosis, previous aortic surgery due to aneurysm, vasculitis, and radiotherapy. Ischemia may be present as only involving the mucosa, which can be managed conservatively, but full-thickness necrosis requires urgent surgical intervention. Endoscopic examination is the gold standard in initial diagnosis. Ischemic gangrene of the rectosigmoid colon is a rare condition and can be life-threatening unless an urgent surgical intervention is performed.

## Introduction

Acute ischemia of the rectosigmoid colon is an extremely rare condition due to rich collateral blood network and can vary between 2% and 5% of all cases of acute ischemic colitis [1-3]. Ischemic proctitis may be caused by acute vascular occlusion, such as after aortic surgical and radiological intervention, severe vascular disease, a low flow state, radiotherapy, and vasculitis [4,5].

Herein, we present the case of a male patient who completely recovered from local advanced rectal cancer (cT3N positive) following total neoadjuvant treatment (TNT) [6] and was in the watch-and-wait (WW) protocol [7], but later developed acute ischemic proctosigmoiditis.

## Case presentation

**Figure 1:**
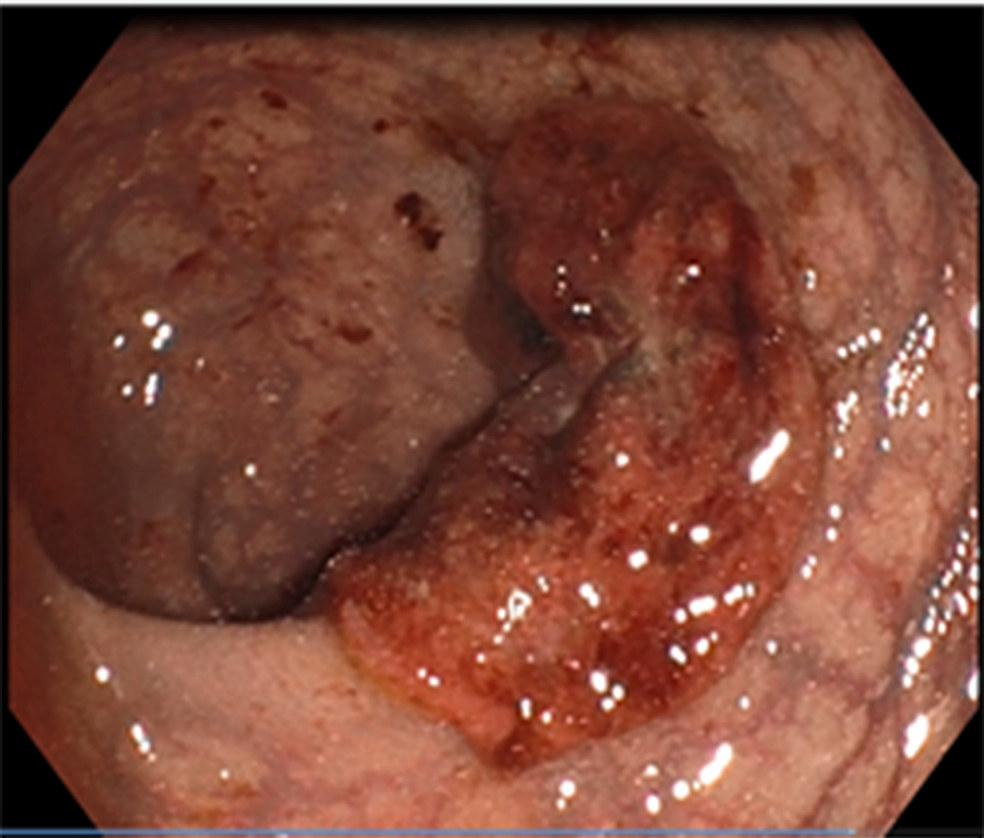
Endoscopic appearance of the patient with rectal cancer before the chemoradiotherapy treatment.

**Figure 2:**
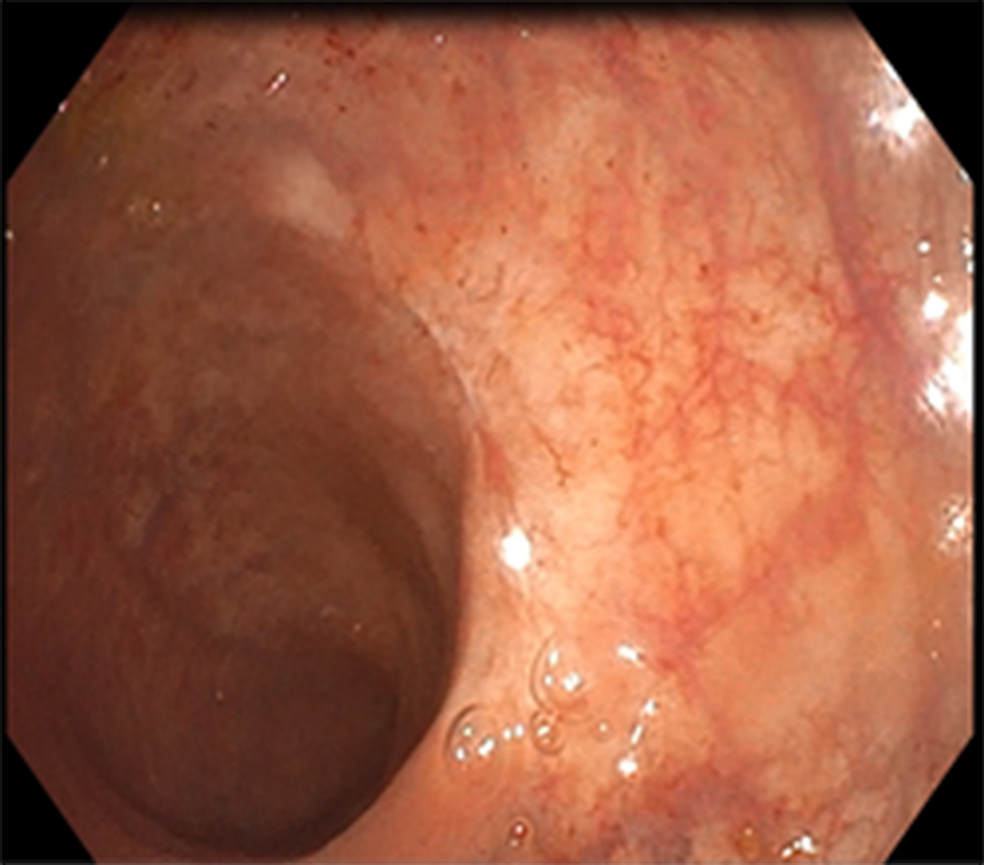
Endoscopic appearance of the patient with rectal cancer after chemoradiotherapy treatment.

A 58-year-old male patient was admitted to our clinic with complaints of abdominal pain, fever, weakness, and bloody stool. An initial assessment revealed the following vital parameters: blood pressure of 110/70 mmHg, pulse of 80 beats/minute, respiratory rate of 20 breaths/minute, saturation of 97% on room air, and temperature of 38°C. The patient had a local advanced rectal cancer history, which achieved a complete clinical response (cCR) after TNT, and he was one of our patients in the WW program (Figures 1, 2). The patient received pelvic radiotherapy dose of 50.4/Gy delivered in 28 fractions and concomitant oral capecitabin 825 mg/m^2^ twice daily during radiotherapy. After four weeks, TNT was administered, and the consolidation chemotherapy regimen was oxaliplatin 130 mg/m^2^ on day 1 plus capecitabine 1,000 mg/m^2^ twice daily on days 1-14 every three weeks for eight cycles. In the patient, the time between cCR and developing of acute ischemia was 14 months.

**Figure 3:**
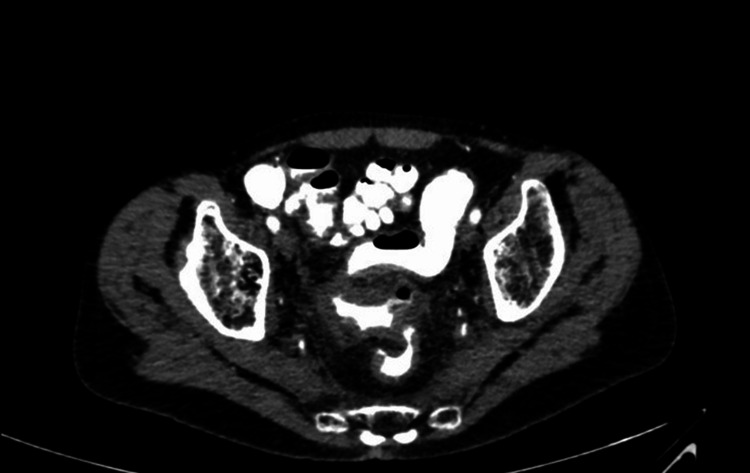
Rectosigmoid colon wall thickening and pneumatosis on the rectal wall.

**Figure 4:**
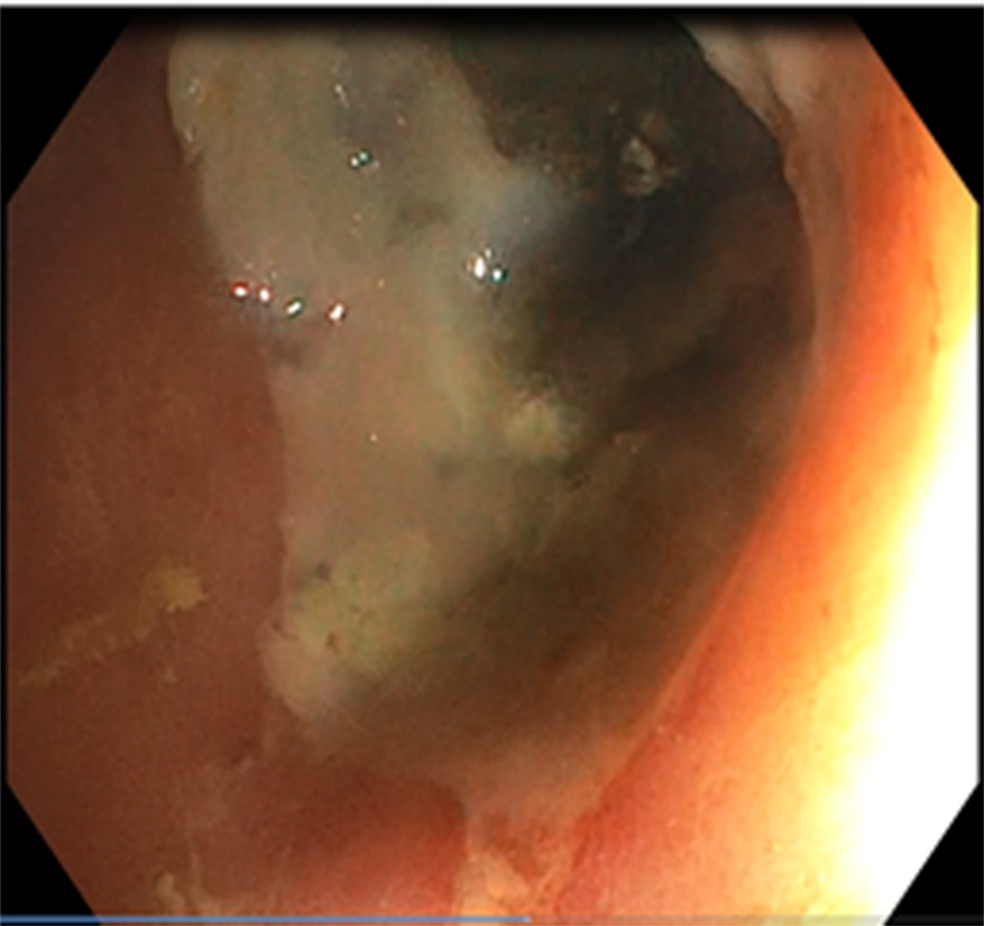
Distal part of the rectal mucosa in normal appearance.

**Figure 5:**
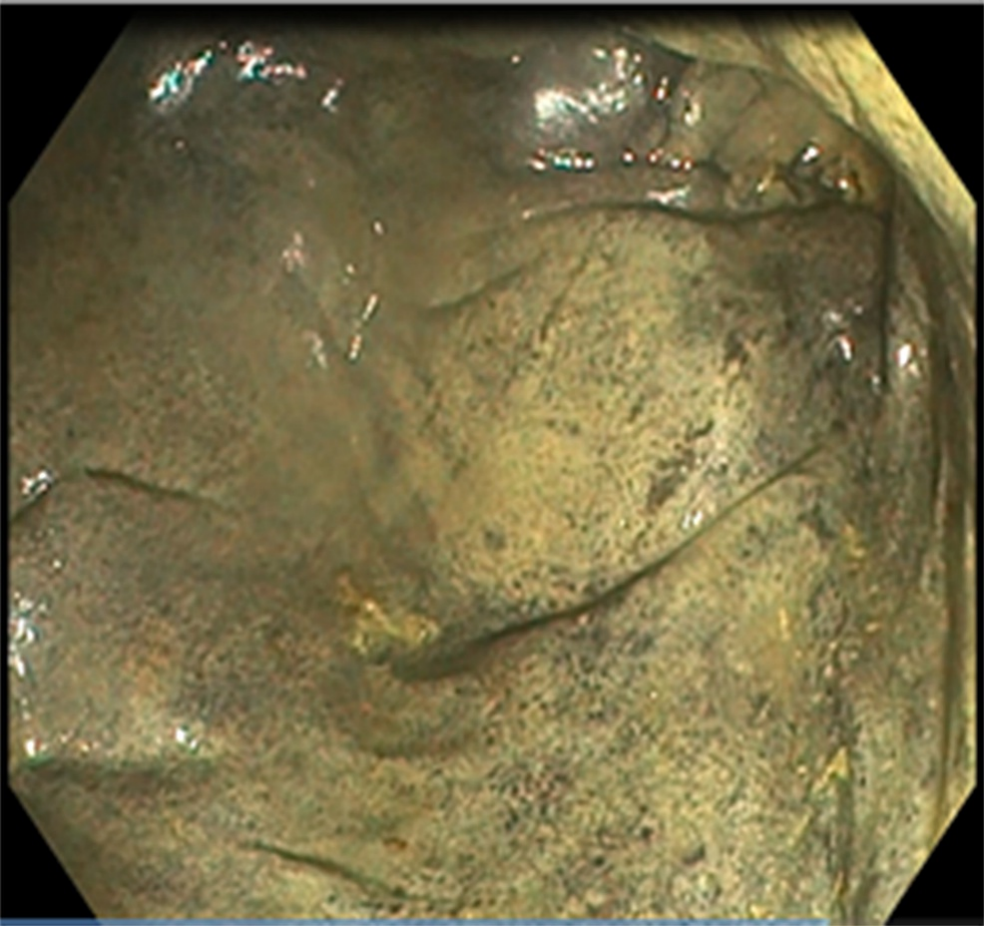
Ischemic appearance of the mucosa extends from the mid-rectum to the distal sigmoid colon.

**Figure 6:**
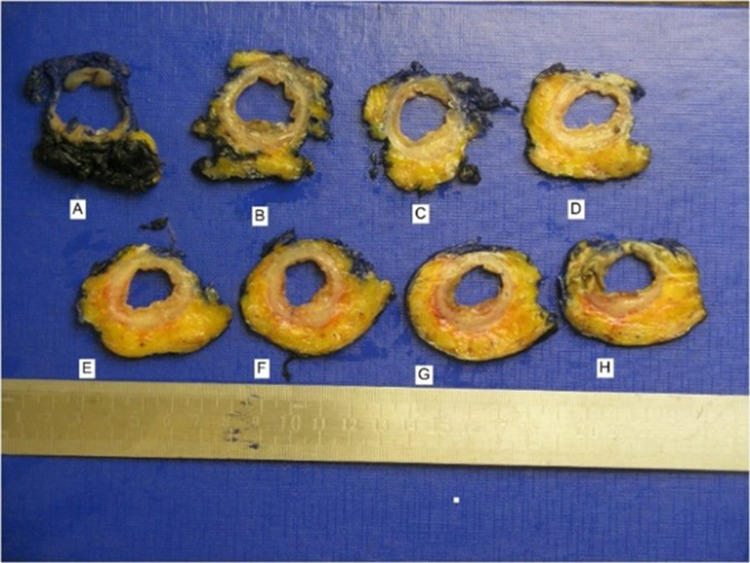
Pathology examinations revealed full-thickness ischemia of the rectosigmoid colon.

**Figure 7:**
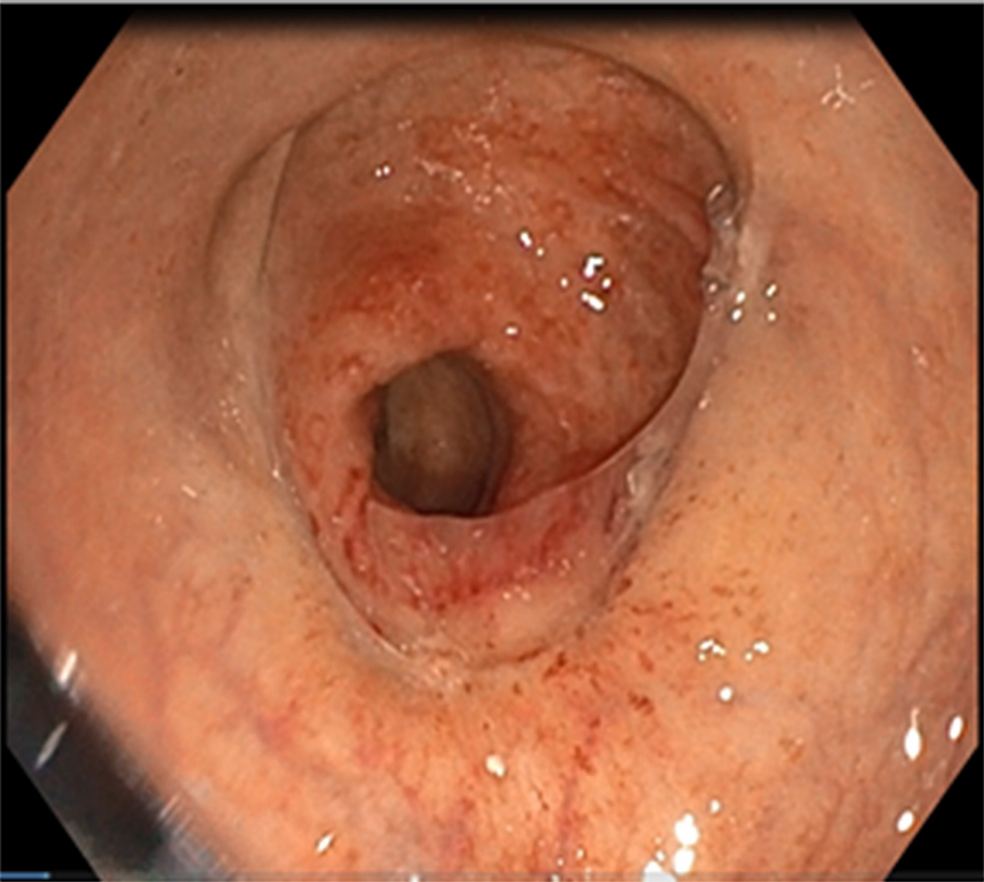
Postoperative control rectosigmoidoscopy after one year showed normal anastomotic line.

Laboratory tests showed an elevated white blood cell (WBC) count of 14,000/μL, neutrophil count of 7,240/ μL, and C-reactive protein level of 23 mg/ μL. Abdominal computed tomography (CT) revealed concentric and irregular thickening of the rectosigmoid colon with stranding of the adjacent fat from up to the mid-rectum and distal sigmoid colon, and signs of air bubbles in the colonic wall (Figure 3). Flexible rectosigmoidoscopy showed mucosal ischemia beginning in the middle of the rectum and continuing about 20 cm to the proximal site of the colon segment (Figures 4, 5). Intravenous fluid, ciprofloxacin, and metronidazole were started immediately. Laparoscopic Low anterior resection (LAR) and protective ileostomy were performed. His pathologic reports revealed mural and transmural infarction on the colonic wall. No tumor cells were observed (Figure 6). His postoperative period was uneventful, and he was discharged from the hospital after three postoperative days. Ileostomy closure was performed after four weeks of surgery, and he was discharged after two days. His control colonoscopy after one year revealed normal signs (Figure 7).

## Discussion

Ischemic colitis is the most common form of gastrointestinal ischemia, accounting for 50% to 60% of all cases and occurring with an incidence of 4.5 to 44 cases per 100,000 person-years [3].

The underlying mechanism leading to ischemic proctosigmoiditis is not completely understood.

Bharucha et al. [4] reviewed all ischemic proctosigmoiditis cases between 1976 and 1991 at Mayo Clinic and identified 10 cases (six acute and four chronic cases) out of 328 cases of colonic ischemia. Most cases of ischemic proctitis were secondary to radiotherapy, previous vascular intervention, aortoiliac surgery, vasculitis, or myointimal hyperplasia of the mesenteric vein [8-10].

The clinical presentation of patients with ischemic proctosigmoiditis may be similar to those with inflammatory bowel disease or pseudomembranous colitis and lead to misdiagnosis [11]. Histology is often required to confirm the diagnosis. Endoscopy should be the initial diagnostic tool, which can confirm the presence of mucosal ischemia and ulcerations in the rectum. Subsequently, abdominal CT scan should be performed. CT findings such as pneumatosis in the rectal wall or extraluminal air are suggestive of transmural gangrene and requires urgent surgery. It has been shown that delayed interventions in acute ischemia of the rectum can leads to high mortality and morbidity even after surgical resection [12,13]. The location and length of the ischemic segment determine the type of operation (such as Hartman or anterior resection of the rectum).

Herein, we reported acute ischemic proctosigmoiditis in rectal cancer patients in the WW protocol. To the best of our knowledge, this is the first ever reported case among the WW protocol patients. The patient was in our close follow-up, and his two months earlier endoscopic examination was normal. The cause of ischemia in our patient is unclear. We conjecture that previous radiotherapy may cause this condition. Since the distal part of the rectal mucosa appearance seemed healthy, we performed laparoscopic LAR and protective ileostomy, with the latter closure being performed four weeks later. Our patient is feeling healthy and his quality of life is pretty good.

## Conclusions

Ischemic gangrene of the rectosigmoid colon is a rare emergency condition. Ischemic gangrene of the rectum may be rarely seen in patients with rectal cancers after radiotherapy. Endoscopic and CT examination is necessary for diagnosis and treatment options. Immediate surgical intervention is inevitable in full-thickness ischemia of the rectosigmoid colon.
